# 4-[4-(1*H*-Imidazol-4-yl)phen­yl]-1*H*-imidazole

**DOI:** 10.1107/S1600536812032485

**Published:** 2012-08-01

**Authors:** Qian Wang, Xiao-Li Zhao

**Affiliations:** a Shanghai Key Laboratory of Green Chemistry and Chemical Processes, Department of Chemistry, East China Normal University, 3663 North Zhongshan Road, Shanghai 200062, People’s Republic of China

## Abstract

In the mol­ecule of the title compound, C_12_H_10_N_4_, the two imidazole substituents are related by inversion symmetry and each forms a dihedral angle of 25.02 (8)° with the benzene ring. In the crystal, mol­ecules are linked through N—H⋯N hydrogen bonds, forming cyclic units [graph-set *R*_4_^4^(28)], which generate a layered structure extending across (011).

## Related literature

For the synthesis of the title compound, see: Petersen (1950 ▶); Huisman (1997 ▶); Have (1997 ▶). For a similar structure, see: Gao & Duan (2012 ▶). For graph-set analysis, see: Etter *et al.* (1990 ▶).
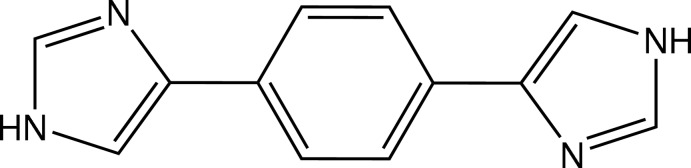


## Experimental

### 

#### Crystal data

C_12_H_10_N_4_
*M* *_r_* = 210.24
Orthorhombic, 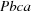

*a* = 6.8604 (2) Å
*b* = 9.4534 (3) Å
*c* = 16.4789 (6) Å
*V* = 1068.72 (6) Å^3^
*Z* = 4
Mo *K*α radiation
μ = 0.08 mm^−1^
*T* = 296 K
0.40 × 0.35 × 0.30 mm

#### Data collection

Bruker SMART CCD area-detector diffractometer
Absorption correction: multi-scan (*SADABS*; Sheldrick, 1996 ▶) *T*_min_ = 0.967, *T*_max_ = 0.975
11170 measured reflections
932 independent reflections
746 reflections with *I* > 2σ(*I*)
*R*_int_ = 0.035

#### Refinement

*R*[*F* ^2^ > 2σ(*F* ^2^)] = 0.032
*wR*(*F* ^2^) = 0.096
*S* = 1.07
932 reflections
77 parameters
H atoms treated by a mixture of independent and constrained refinement
Δρ_max_ = 0.11 e Å^−3^
Δρ_min_ = −0.13 e Å^−3^

### 

Data collection: *SMART* (Bruker, 2001 ▶); cell refinement: *SAINT* (Bruker, 2001 ▶); data reduction: *SAINT*; program(s) used to solve structure: *SHELXS97* (Sheldrick, 2008 ▶); program(s) used to refine structure: *SHELXL97* (Sheldrick, 2008 ▶); molecular graphics: *SHELXTL* (Sheldrick, 2008 ▶); software used to prepare material for publication: *SHELXTL* and local programs.

## Supplementary Material

Crystal structure: contains datablock(s) I, global. DOI: 10.1107/S1600536812032485/zs2214sup1.cif

Structure factors: contains datablock(s) I. DOI: 10.1107/S1600536812032485/zs2214Isup2.hkl

Supplementary material file. DOI: 10.1107/S1600536812032485/zs2214Isup3.cml

Additional supplementary materials:  crystallographic information; 3D view; checkCIF report

## Figures and Tables

**Table 1:** Hydrogen-bond geometry (Å, °)

*D*—H⋯*A*	*D*—H	H⋯*A*	*D*⋯*A*	*D*—H⋯*A*
N1—H1*B*⋯N2^i^	0.981 (17)	1.863 (18)	2.8364 (17)	170.8 (17)

Symmetry code: (i) 
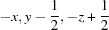
.

**Fig. 1.**
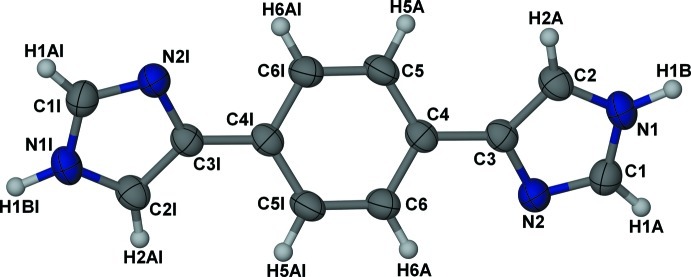
Molecular conformation and atom-numbering scheme for the title compound, with probability ellipsoids drawn at the 50% level. For symmetry code (1): -x, -y + 2, -z.

**Fig. 2.**
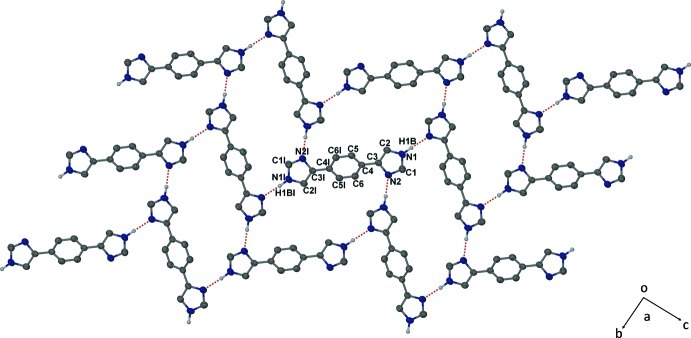
The two-dimensional layered structure of the title compound viewed down a.

## supplementary crystallographic information

### Comment

Intense interest in the chemistry of metal-organic frameworks stems from their intriguing structural features and potential applications in catalysis, adsorption, luminescence, etc. The title compound C_12_H_10_N_4_ was designed and synthesized to enable the construction of metal-organic frameworks because of its versatile coordination modes in respect to metal complexation. In the structure of this compound (Fig. 1), the molecule has inversion symmetry with the two imidazole moieties rotated slightly out of the plane of the benzene ring [dihedral angle, 25.02 (8)°]. In the crystal, the molecules are linked through N—H···N hydrogen bonds (Table 1), forming inter-associated cyclic units [graph set *R*^4^_4_(28)] which generate a two-dimensional layered structure extending across (011) (Fig. 2).

### Experimental

The title compound was synthesized according to literature methods (Petersen, 1950; Huisman, 1997; Have, 1997). A single crystal suitable for the X-ray diffraction study was obtained serendipitously in an attempt to synthesize a La^III^ complex. A mixture of 5-(4-(1*H*-imidazol-5-yl)phenyl)-1*H*-imidazole and lanthanum(III) nitrate hexahydrate in water was subjected to hydrothermal conditions at 85 °C for three days and then cooled to room temperature to give colorless crystals of the title compound.

### Refinement

The hydrogen atom on the N atom was located in a difference-Fourier map and refined isotropically. The other H atoms were positioned with idealized geometry (C—H = 0.93 Å) and allowed to ride, with *U*_iso_(H) = 1.2 *U*_eq_(C).

### Crystal data

**Table:**

C_12_H_10_N_4_	*F*(000) = 440
*M**_r_* = 210.24	*D*_x_ = 1.307 Mg m^−^^3^
Orthorhombic, *P**b**c**a*	Mo *K*α radiation, λ = 0.71073 Å
Hall symbol: -P 2ac 2ab	Cell parameters from 2852 reflections
*a* = 6.8604 (2) Å	θ = 2.5–23.0°
*b* = 9.4534 (3) Å	µ = 0.08 mm^−^^1^
*c* = 16.4789 (6) Å	*T* = 296 K
*V* = 1068.72 (6) Å^3^	Block, colorless
*Z* = 4	0.40 × 0.35 × 0.30 mm

### Data collection

**Table:**

Bruker SMART CCD area-detector diffractometer	932 independent reflections
Radiation source: fine-focus sealed tube	746 reflections with *I* > 2σ(*I*)
Graphite monochromator	*R*_int_ = 0.035
φ and ω scans	θ_max_ = 25.0°, θ_min_ = 3.9°
Absorption correction: multi-scan (*SADABS*; Sheldrick, 1996)	*h* = −8→8
*T*_min_ = 0.967, *T*_max_ = 0.975	*k* = −11→11
11170 measured reflections	*l* = −19→19

### Refinement

**Table:**

Refinement on *F*^2^	Primary atom site location: structure-invariant direct methods
Least-squares matrix: full	Secondary atom site location: difference Fourier map
*R*[*F*^2^ > 2σ(*F*^2^)] = 0.032	Hydrogen site location: inferred from neighbouring sites
*wR*(*F*^2^) = 0.096	H atoms treated by a mixture of independent and constrained refinement
*S* = 1.07	*w* = 1/[σ^2^(*F*_o_^2^) + (0.0539*P*)^2^ + 0.1236*P*] where *P* = (*F*_o_^2^ + 2*F*_c_^2^)/3
932 reflections	(Δ/σ)_max_ < 0.001
77 parameters	Δρ_max_ = 0.11 e Å^−^^3^
0 restraints	Δρ_min_ = −0.13 e Å^−^^3^

### Special details

**Table:**

Geometry. All e.s.d.'s (except the e.s.d. in the dihedral angle between two l.s. planes) are estimated using the full covariance matrix. The cell e.s.d.'s are taken into account individually in the estimation of e.s.d.'s in distances, angles and torsion angles; correlations between e.s.d.'s in cell parameters are only used when they are defined by crystal symmetry. An approximate (isotropic) treatment of cell e.s.d.'s is used for estimating e.s.d.'s involving l.s. planes.
Refinement. Refinement of *F*^2^ against ALL reflections. The weighted *R*-factor *wR* and goodness of fit *S* are based on *F*^2^, conventional *R*-factors *R* are based on *F*, with *F* set to zero for negative *F*^2^. The threshold expression of *F*^2^ > σ(*F*^2^) is used only for calculating *R*-factors(gt) *etc*. and is not relevant to the choice of reflections for refinement. *R*-factors based on *F*^2^ are statistically about twice as large as those based on *F*, and *R*- factors based on ALL data will be even larger.

### Fractional atomic coordinates and isotropic or equivalent isotropic displacement parameters (Å^2^)

**Table:**

	*x*	*y*	*z*	*U*_iso_*/*U*_eq_	
N1	−0.04339 (18)	0.62662 (12)	0.21679 (8)	0.0486 (4)	
N2	0.03252 (17)	0.85297 (11)	0.21277 (7)	0.0465 (3)	
C1	0.0142 (2)	0.74017 (14)	0.25866 (10)	0.0505 (4)	
H1A	0.0387	0.7394	0.3142	0.061*	
C2	−0.0646 (2)	0.66854 (14)	0.13827 (9)	0.0459 (4)	
H2A	−0.1035	0.6125	0.0948	0.055*	
C3	−0.01772 (19)	0.80895 (14)	0.13570 (8)	0.0402 (3)	
C4	−0.01031 (18)	0.90577 (13)	0.06630 (8)	0.0398 (4)	
C5	−0.1213 (2)	0.88440 (13)	−0.00307 (8)	0.0465 (4)	
H5A	−0.2038	0.8065	−0.0059	0.056*	
C6	0.1116 (2)	1.02373 (13)	0.06775 (8)	0.0461 (4)	
H6A	0.1879	1.0407	0.1134	0.055*	
H1B	−0.054 (3)	0.5309 (18)	0.2395 (11)	0.081 (5)*	

### Atomic displacement parameters (Å^2^)

**Table:**

	*U*^11^	*U*^22^	*U*^33^	*U*^12^	*U*^13^	*U*^23^
N1	0.0551 (7)	0.0350 (6)	0.0558 (8)	0.0034 (5)	0.0020 (6)	0.0067 (6)
N2	0.0532 (7)	0.0382 (6)	0.0483 (7)	−0.0007 (5)	−0.0064 (5)	0.0016 (5)
C1	0.0568 (9)	0.0458 (8)	0.0490 (9)	0.0034 (7)	−0.0077 (6)	0.0040 (7)
C2	0.0524 (8)	0.0376 (7)	0.0477 (9)	−0.0004 (6)	0.0043 (6)	−0.0035 (6)
C3	0.0402 (7)	0.0346 (7)	0.0458 (8)	0.0014 (5)	0.0024 (6)	−0.0020 (6)
C4	0.0423 (7)	0.0327 (7)	0.0442 (8)	0.0004 (5)	0.0044 (6)	−0.0033 (5)
C5	0.0523 (8)	0.0383 (7)	0.0488 (8)	−0.0125 (6)	0.0001 (6)	−0.0032 (6)
C6	0.0504 (8)	0.0440 (7)	0.0441 (8)	−0.0093 (6)	−0.0029 (6)	−0.0017 (6)

### Geometric parameters (Å, º)

**Table:**

N1—C1	1.3359 (17)	C2—H2A	0.9300
N1—C2	1.3611 (19)	C3—C4	1.4657 (18)
N1—H1B	0.981 (17)	C4—C5	1.3883 (19)
N2—C1	1.3133 (17)	C4—C6	1.3940 (18)
N2—C3	1.3801 (18)	C5—C6^i^	1.3766 (17)
C1—H1A	0.9300	C5—H5A	0.9300
C2—C3	1.3665 (19)	C6—H6A	0.9300
			
C1—N1—C2	106.79 (12)	C2—C3—C4	129.71 (12)
C1—N1—H1B	124.5 (11)	N2—C3—C4	121.40 (11)
C2—N1—H1B	128.5 (11)	C5—C4—C6	117.36 (12)
C1—N2—C3	105.14 (11)	C5—C4—C3	122.18 (12)
N2—C1—N1	112.54 (14)	C6—C4—C3	120.46 (12)
N2—C1—H1A	123.7	C6^i^—C5—C4	121.28 (12)
N1—C1—H1A	123.7	C6^i^—C5—H5A	119.4
N1—C2—C3	106.67 (12)	C4—C5—H5A	119.4
N1—C2—H2A	126.7	C5^i^—C6—C4	121.36 (12)
C3—C2—H2A	126.7	C5^i^—C6—H6A	119.3
C2—C3—N2	108.86 (12)	C4—C6—H6A	119.3
			
C3—N2—C1—N1	0.22 (16)	N2—C3—C4—C5	−156.22 (12)
C2—N1—C1—N2	−0.19 (16)	C2—C3—C4—C6	−153.60 (14)
C1—N1—C2—C3	0.07 (15)	N2—C3—C4—C6	24.22 (18)
N1—C2—C3—N2	0.06 (15)	C6—C4—C5—C6^i^	−0.1 (2)
N1—C2—C3—C4	178.10 (13)	C3—C4—C5—C6^i^	−179.71 (13)
C1—N2—C3—C2	−0.17 (15)	C5—C4—C6—C5^i^	0.1 (2)
C1—N2—C3—C4	−178.40 (12)	C3—C4—C6—C5^i^	179.72 (12)
C2—C3—C4—C5	26.0 (2)		

Symmetry code: (i) −*x*, −*y*+2, −*z*.

### Hydrogen-bond geometry (Å, º)

**Table:**

*D*—H···*A*	*D*—H	H···*A*	*D*···*A*	*D*—H···*A*
N1—H1*B*···N2^ii^	0.981 (17)	1.863 (18)	2.8364 (17)	170.8 (17)

Symmetry code: (ii) −*x*, *y*−1/2, −*z*+1/2.
